# A Review of the Parameters Controlling Crack Growth in AM Steels and Its Implications for Limited-Life AM and CSAM Parts

**DOI:** 10.3390/ma19020372

**Published:** 2026-01-16

**Authors:** Rhys Jones, Andrew Ang, Nam Phan, Michael R. Brindza, Michael B. Nicholas, Chris Timbrell, Daren Peng, Ramesh Chandwani

**Affiliations:** 1 ARC Industrial Transformation Training Centre on Surface Engineering for Advanced Materials, School of Engineering, Swinburne University of Technology, John Street, Hawthorn, VIC 3122, Australia; aang@swin.edu.au (A.A.); daren.peng@monash.edu (D.P.); 2 Department of Mechanical and Aerospace Engineering, Monash University, Clayton, VIC 3800, Australia; 3 Structures Division, Naval Air Systems Command, Patuxent River, MD 20670, USA; 4 Air Warfare & Weapons Department, Air Platforms Division, Office of Naval Research, Arlington, VA 22203-1995, USA; 5 US Army Research Laboratory, U.S. Army Combat Capabilities Development Command Weapons and Materials Research Directorate, Aberdeen Proving Ground, Aberdeen, MD 20852, USA; 6 Zentech International Limited, 590B Finchley Road, London NW11 7RX, UK; chris.timbrell@zentech.co.uk (C.T.); ramesh.chandwani@zentech.co.uk (R.C.)

**Keywords:** additively manufactured steels, CSAM 316L steel, fatigue crack growth, fracture mechanics, limited-life replacement parts, build quality

## Abstract

This paper reviews the fracture mechanics parameters associated with the variability in the crack growth curves associated with forty-two different tests that range from additively manufactured (AM) steels to cold spray additively manufactured (CSAM) 316L steel. As a result of this review, it is found that, to a first approximation, the effects of different building processes and *R*-ratios on the relationship between Δ*K* and the crack growth rate (*da/dN*) can be captured by allowing for changes in the fatigue threshold and the apparent cyclic toughness in the Schwalbe crack driving force (Δ*κ*). Whilst this observation, when taken in conjunction with similar findings for AM Ti-6Al-4V, Inconel 718, Inconel 625, and Boeing Space Intelligence and Weapon Systems (BSI&WS) laser powder bed (LPBF)-built Scalmalloy^®^, as well as for a range of CSAM pure metals, go a long way in making a point; it is NOT a mathematical proof. It is merely empirical evidence. As a result, this review highlights that for AM and CSAM materials, it is advisable to plot the crack growth rate (*da/dN*) against both Δ*K* and Δ*κ*. The observation that, for the AM and CSAM steels examined in this study, the *da/dN* versus Δ*κ* curves are similar, when coupled with similar observation for a range of other AM materials, supports a prior study that suggested using fracture toughness measurements in conjunction with the flight load spectrum and the operational life requirement to guide the choice of the building process for AM Ti-6Al-4V parts. The observations outlined in this study, when taken together with related findings given in the open literature for AM Ti-6Al-4V, AM Inconel 718, AM Inconel 625, and BSI&WS LPFB-built Scalmalloy^®^, as well as for a range of CSAM-built pure metals, have implications for the implementation and certification of limited-life AM parts.

## 1. Introduction

The United States (US) Department of Defence (DoD) memo [1] mandates the use of additive manufacturing (AM) within the US DoD. The US Army Directive 2019-19 [2] states the following:

(i) AM can be used to address the operational issues that can arise due to parts obsolescence, logistics, and sustained operations;
(ii) AM has the potential to transform battlefield logistics through on-demand fabrication of parts close to the point of need;
(iii) AM has the potential to reduce the large number of parts that are currently stored and that need to be transported to where they are required.

Similar statements can be found in the US Navy study [3]. A review paper [4] noted that whilst additive manufacturing offers the potential to rapidly print customised parts, there is a need for their mechanical behaviour to be better understood. The United States Air Force (USAF) Structures Bulletin EZ-SB-19-01 [5] subsequently stated that one of the most difficult challenges facing the certification of AM parts is the ability to predict their durability and damage tolerance (DADT).

The USAF Structures Bulletin EZ-SB-19-01 [5] also states that the DADT assessment of an AM aircraft part requires a linear elastic fracture mechanics analysis. In this context, it has long been known [6] that, for long cracks in conventionally manufactured metals, the relationship between the crack growth rate (*da/dN*) and the change in the stress intensity factor per cycle Δ*K* (=*K_max_* − *K_min_*, where *K_max_* and *K_min_* are the maximum and minimum values of the stress intensity factor (*K*) in a load cycle) can be dependent on the microstructure, and that changes in the processing conditions, i.e., heat treatments, etc., can produce quite different microstructures. It is thus no surprise that, as discussed in [7,8,9], different AM building processes and different post-build processes can result in microstructures that can yield quite different, long-crack *da/dN* versus Δ*K* curves.

The problem is compounded by the fact that the *da/dN* versus Δ*K* curves associated with AM materials can exhibit a large degree of variability as well as anisotropy [10]. Furthermore, as noted in the USAF Structures Bulletin EZ-SB-19-01 [5] and in the USAF airworthiness certification standard MIL-STD-1530D [11], it is essential that this variability be accounted for in the DADT assessment. This is particularly important when performing the Risk of Failure analysis that is required by the US Joint Services Structural Guidelines JSSG2006 [12].

In this context, the AGARD Round-Robin test programme, AGARD-R-732 [13], is acknowledged as being amongst the first studies to highlight the variability in the crack growth curves seen by naturally occurring cracks in conventionally built metals. (This study focused on the aluminium alloy AA2024-T3.) The subsequent paper by Newman et al. [14] led to the belief that the worst-case (upper-bound) *da/dN* versus Δ*K* curves that are needed for a durability assessment corresponded to the “crack-closure” free *da/dN* versus Δ*K* curve. It was only recently that the mistake in [14], which led to this proposition, was discovered [15]. Fortunately, it is now known [16] that the worst-case *da/dN* versus ΔK curves associated with the AGARD Round Robin and other companion studies presented in AGARD-R-762 [17] and in [18,19,20] can be predicted using the Hartman–Schijve crack growth equation [21] for the growth of long cracks in AA2024-T3, albeit with the fatigue threshold term set to a small (near zero) value.

To clarify this statement, it should be recalled that the Hartman–Schijve crack growth equation can be written in the following form:

*da/dN* = *D* (Δ*κ*)^*p*^ (1)

where *D* and *p* are material constants [21]. The term Δ*κ* in Equation (1) is the Schwalbe crack driving force [22,23], which can be written as follows:

Δ*κ* = [(Δ*K* − Δ*K_thr_*)/√(1 − *K_max_*/*A*)] (2)

The term Δ*K_thr_* is the fatigue threshold, and *A* is the apparent cyclic fracture toughness.

The prediction mentioned above for the growth of naturally occurring three-dimensional (3D) cracks in the AA2024-T3 AGARD Round Robin study used the values of *D*, *p*, and *A* obtained from long-crack tests on AA2024-T3 with the fatigue threshold term Δ*K_thr_* set to 0.1 MPa √m. This approach is consistent with the statement in Appendix X3 of the fatigue test standard ASTM E647-23b [24] that “It is not clear if a measurable threshold exists for the growth of small fatigue cracks”. It is also consistent with observations given in the USAF Durability Design Handbook [25], the USAF Risk Analysis documentation [26], and in [27,28,29], which state/imply that cracks in conventionally manufactured aerospace parts grow from day one, i.e., from the day that the airframe enters service. There is no reason to believe that this experience would not also be seen by AM parts in operational aircraft, particularly since the build quality associated with AM parts can be expected to be less than that associated with conventionally built aerospace quality parts [3].

Fortunately, it is now known [10,15,16,30,31,32,33,34,35,36,37,38,39,40,41,42,43,44,45,46,47,48,49,50,51,52,53,54] that Equations (1) and (2) can often be used to represent the growth of cracks in a range of both AM and cold spray additively manufactured materials and that the variability and anisotropy can often be accounted for by allowing for the effect of the build, and the post-processing protocol, on just two fracture mechanics parameters, namely Δ*K_thr_* and *A*. (Here, it should be noted that one purpose of the present review is to evaluate if these observations can be extended to AM steels).

At this point, it should be noted that, as discussed in [55], the parameters Δ*K_thr_* and *A* are best thought of as “fitting” parameters. Nevertheless, despite these parameters being obtained using an optimisation approach, the resultant long-crack equation with the fatigue threshold (Δ*K_thr_*) set to a small, near-zero value has been shown to often predict the growth of naturally occurring three-dimensional cracks in a range of both conventionally built and AM metals (see [15,16,21,30,31,33,35,36,43,45,56,57,58,59,60,61,62,63]). The important word in this sentence is the word “predict”. This is important since a predictive capability is central to the airworthiness certification of an AM/CSAM part. This predictive capability is aptly illustrated in [21], which analysed an early F-111 wing failure, and in [45], which analysed failure in a US Navy F/A-18 centre barrel fatigue test. In both instances the tests articles were subjected to a representative operational flight load spectrum.

This ability of Equations (1) and (2) to accurately predict the growth of small cracks in AM specimens is aptly illustrated in [16], where

(i) The previously published Hartman–Schijve equation for long cracks in conventionally manufactured and AM Inconel 718 [44], with the term Δ*K_thr_* set to be equal to 0.1 MPa √m, was used to (accurately) predict the upper-bound curve associated with the growth of naturally occurring three-dimensional cracks in both electron beam melt (EBM) and selective laser melt (SLM)-built Inconel 718;
(ii) The previously published Hartman–Schijve equation for long cracks in conventionally built CP-Ti, with the term Δ*K_thr_* set to be equal to 0.1 MPa √m, was used to (accurately) predict the growth of naturally occurring three-dimensional cracks in WAAM CP-Ti [16].

Similarly, Equations (1) and (2), with the term Δ*K_thr_* set to be equal to 0.1 MPa √m, have also been shown [15,35] to predict the growth of naturally occurring 3D cracks in Boeing Space Intelligence and Weapon Systems’ (BSI&WS) laser-powder-fusion-built Scalmalloy^®^, which is an AM aluminium alloy. Here, it should be noted that in [35], the Scalmalloy^®^ specimens had, prior to fatigue testing, been exposed for twenty-eight days in an ASTM Standard B117-19 environmental chamber [64] to a 5% (by weight) salt fog at 35 °C. Equations (1) and (2), with the term Δ*K_thr_* set to be equal to 0.1 MPa √m, have also been shown [36] to predict the growth of naturally occurring cracks in AM Ti-6Al-4V.

The Hartman–Schijve equation has also been shown to be able to account for crack growth in adhesives [65], polymers [66], nanocomposites [67], and fibre-reinforced plastic composites [68,69,70,71,72,73]. Variants of this formulation can be found in [74,75,76,77,78,79,80,81,82,83,84,85].

An advantage of this formulation is that it is now commercially available, via the Zencrack^®^ software interface [86], in the general-purpose finite element programmes Abaqus^®^, NASTRAN^®^, and ANSYS^®^. This software also contains an automated computer programme for determining the constants in the Hartman–Schijve crack growth equation. As such, this formulation is now commercially available for assessing the DADT of AM parts.

As a result, one object of this review is to evaluate if the variability in the long crack *da/dN* versus Δ*K* curves seen in tests on AM steels can also be captured by allowing for changes in the fatigue threshold (Δ*K_thr_*) and the apparent cyclic toughness (*A*) in the Schwalbe crack driving force (Δ*κ*). However, it should be stressed that whilst the large number of examples given in this paper, and in prior studies on AM Ti-6Al-4V, AM Inconel 625, AM Inconel 718, AM CP-Ti, and Scalmalloy^®^, go a long way to making a point, they are NOT mathematical proofs. They are merely empirical evidence. Nevertheless, the evidence is quite compelling. The implications of these observations for the use of AM and CSAM to build limited-life replacement parts and parts for attritable aircraft will also be discussed.

Although not stated above, USAF Structures Bulletin EZ-SB-19-01 [5] also requires the fatigue thresholds to be determined. In the case of a durability analysis of an AM/CSAM part, this means that an estimate is needed for the fatigue threshold associated with naturally arising 3D cracks that nucleate from porosity/lack of fusion or corrosion pits. This statement is true regardless of whether the part is built from AM steel, LPBF Scalmalloy^®^, AM Ti-6Al-4V, AM Inconel 718, etc.

This leads to the question of how to compute the intensity factor for an AM part that contains a naturally occurring 3D crack that has nucleated from either a surface-breaking or a near-surface manufacturing defect, such as porosity/lack of fusion, or from a corrosion pit in an AM part.

In this context, it should be noted that many authors, when attempting to model this class of naturally occurring 3D cracks, have treated them as surface-breaking, semi-elliptical cracks [87,88,89] and then used the approximate stress intensity factor solutions (for a semi-elliptical surface crack) given by Newman and Raju [90], rather than modelling the geometry of the defect/lack of porosity/etc and the crack separately, as was performed in [16,30]. Other researchers [91,92,93,94,95,96,97] have used the Murakami approximation [98] to estimate the stress intensity factor at the base of the crack. The way in which the Murakami approximation is used for a crack that emanates from the base of a surface-breaking manufacturing defect, a corrosion pit, or from the base of surface-breaking porosity/lack of fusion in a large structure subjected to uniform loading is that the maximum value of the stress intensity factor (*K_max_*), which arises at the deepest point of the crack, is approximated using the following formula:

*K_max,Murakami_* = 0.65 σ × √(π(√Area)) (3)

Here, σ is the remote stress, and the term “Area” in Equation (3) is the cross-sectional area of the defect (surface-breaking porosity/lack of fusion or pit) plus the area of the small naturally occurring 3D crack. The suffix Murakami is used to differentiate this approximation from the actual value of *K_max_*. Unfortunately, the ability of these approaches to accurately determine the value of *K* associated with small 3D cracks that nucleate from the base of surface-breaking porosity/lack of fusion in an AM part has not, as yet, been addressed. This paper addresses this shortcoming.

## 2. Materials and Methods

Before discussing crack growth in AM steels, it should be noted that, when discussing the fatigue performance of bridge steels, the US Department of Transportation Federal Highway Administration’s report [99] states the following:

“The insignificance of steel type and weld metal on fatigue resistance greatly simplifies the development of fatigue design rules since it eliminates the need to generate data for every type of structural steel.”

This observation was independently supported in [56], where it was shown that the *da/dN* versus Δ*K* curves associated with five different bridge steels that were tested at a range of *R* ratios essentially fell onto a single-curve *da/dN* versus Δ*κ* curve. It was also found [100] that crack growth in each of these steels could be represented using Equations (1) and (2), allowing for changes in the fatigue threshold (Δ*K_thr_*) and the apparent cyclic toughness (*A*). Interestingly, [100] also revealed that the *da/dN* versus Δ*κ* curve associated with these various steels had similar values of *D* and *p* as the high-strength aerospace steel 4340, viz: *D* = 1.5 × 10^−10^ and *p* = 2. These values of *D* and *p* are also similar to those of the high-strength aerospace steel D6ac, for which *D* = 2.0 × 10^−10^ and *p* = 2 [21]. Here, it should be noted that the yield stresses of 4340 and D6ac steels, which are both above 1500 MPa, are very much greater than those of bridge steels, for which the yield stress is typically of the order of 350 MPa. It was also shown [56] that crack growth in five different cast steels, with yield stresses that varied from 300 MPa to approximately 1000 MPa, also fell onto essentially the same curve. In other words, the crack growth curves associated with this range of conventionally manufactured steels, which had very different chemical compositions and yield stresses, had very similar *da/dN* versus Δ*κ* curves.

With this in mind, the present paper investigates the *da/dN* versus Δ*K* curves associated with a range of AM steels, viz:

(i) 18Ni 300 Maraging steel built using selective laser melt (SLM) and tested at *R* = 0.0, 0.3, and 0.6 [101]. These tests used the ASTM E647 standard [24] compact tension (CT) specimens. Since the heat treatment was not mentioned in [101], it is assumed that the material was in the as-built state.
(ii) Wire and arc additively manufactured (WAAM) 304L steel tested at *R* = 0.1 and both with and without heat treatment [102]. These tests used ASTM E647 standard compact tension (CT) specimens;
(iii) The *R* = 0.1 *da/dN* versus Δ*K* curve given in [103] for a Directed Energy Deposition (DeD) built 304L steel in the as-built condition. These tests used ASTM E647 standard compact tension (CT) specimens;
(iv) The *R* = 0.1 crack growth curve for AM 316L specimens built using selective laser melt (SLM), the crack either parallel or perpendicular to the build direction [104]. Whilst both as-built and heat-treated specimens were tested, their *da/dN* versus Δ*K* curves were essentially identical. In the (subsequent) analyses, these tests are referred to as SLM 316L HIPed. These tests used ASTM E647 standard compact tension (CT) specimens;
(v) The *R* = 0.1 *da/dN* versus Δ*K* curve given in [104] for an as-built SLM 316L specimen. This test, which is labelled SLM as-built, also used ASTM E647 standard compact tension (CT) specimens;
(vi) The *R* = 0.1 *da/dN* versus Δ*K* curves given in [105] for as-built AM 316L steel specimens printed using Laser-Engineered Net Shape (LENS). Specimens with the crack both parallel and perpendicular to the build direction were tested. These specimens are labelled “LENS 316L as-built parallel” and “LENS 316L as-built perpendicular”. These tests used ASTM E647 standard compact tension (CT) specimens.
(vii) The *R* = 0.1 *da/dN* versus Δ*K* curves presented in [106] for ASTM E647 standard compact tension (CT) tests on heat-treated laser additively manufactured (LAM) AerMet 100. This study examined three different heat treatments. The heat treatment procedures resulted in three kinds of heat-treated microstructures, viz:
  (a) Coarse Grain Tempered Martensite microstructure (CG-TM);
  (b) Fine Grain Tempered Martensite microstructure (FG-TM);
  (c) Fine Grain Tempered Martensite microstructure with High contents of Retained Austenite ((FG-TM-HRA).
(viii) The *R* = 0.1 *da/dN* versus Δ*K* curves given in the paper by Nezhadfar et al. [107] for ASTM E647 standard compact tension (CT) specimen tests on LPBF 17-4PH steel. This paper examined two different heat treatments.
(ix) The *R* = 0.1, 0.4, 0.7, and *K*_max_ *da/dN* versus Δ*K* curves given in [108] for ASTM E647 standard compact tension (CT) specimen tests on LPBF 17-4PH in the as-built condition, and the *R* = 0.1, 0.7, and *K*_max_ *da/dN* versus Δ*K* curves for LPBF 17-4PH specimens after heat treatment (HT). This study used a range of different pre-cracking test protocols. The notation associated with each of these various tests is given in Table 1.
(x) The *R* = 0.1, 0.2, and 0.5 *da/dN* versus Δ*K* curves given in [109] for ASTM E647 standard compact tension (CT) tests [24] on as-built WAAM super duplex stainless steel (SDSS) specimens built by AML3D^®^. The specimens were pre-cracked under tension loading. (Unless stated, all of the specimen tests examined in this paper were performed in this fashion.) The notation LM stands for specimens cut with the length direction coinciding with the build direction. The notation TM stands for specimens that were cut transverse to the build direction. The specimens were left in the as-built state; i.e., they were not heat-treated.
(xi) Crack growth in cold spray additively manufactured (CSAM) 316L in both the as-sprayed and the annealed condition [41] tested at *R* = −1. These tests included specimens in the LS and LT directions and in both the as-printed and annealed states. In [41] these, various tests were labelled: As-sprayed 02 LS, As-sprayed 03 LS, As-sprayed 09 LT, As-sprayed 10 LT, Annealed 02, Annealed 03 LS, Annealed 08 LT, and Annealed 09 LT. Unlike all of the other tests evaluated, which all used ASTM E647 standard test specimens, this study used small single-edge notch tension specimens.
(xii) The small crack *da/dN* versus Δ*K* curve given in [30] for WAAM 18Ni 250 Maraging steel. These tests used plain (un-notched) heat-treated specimens, and NOT ASTM 647 CT specimens. In these studies, the cracks were allowed to nucleate naturally.

**Table 1:** Values of Δ*K_thr_* and *A* used in Figure 1 and Figure 2.

Specimen ID, as Shown in Figure 1 and Figure 2	*R*	Δ*K_thr_* (MPa √m)	*A* (MPa √m)
Short crack in heat-treated WAAM 18Ni 250 Maraging steel [30] (thickness = 6.35 mm)	0.1	0.1	220.0
CSAM 316L [41] (thickness = 4.0 mm)			
CSAM 316L as sprayed 02, LS	−1	4.65	34.0
CSAM 316L as sprayed 03, LS	−1	4.9	30.0
CSAM 316L as sprayed 08, LT	−1	3.45	19.5
CSAM 316L as sprayed 09, LT	−1	3.58	18.6
CSAM 316L annealed 09, LT	−1	2.97	38.0
CSAM 316L annealed 10, LT	−1	3.0	38.0
SLM 18Ni 300, in the as-built state [101]	0.05	2.0	78.0
Ibid	0.3	2.0	78.0
Ibid	0.6	0.1	78.0
WAAM 304L [102] (thickness = 3.81 mm)			
WAAM 304L Vertical As printed [102]	0.1	9.8	112.0
WAAM 304L Horizontal As printed [102]	0.1	7.8	85.0
WAAM 304L Vertical Stress relieved [102]	0.1	1.0 (was 3)	22.0
WAAM 304L Horizontal Stress relieved [102].	0.1	2.0 (was 4)	19.0
DED 304, as-built [103] (thickness = 6.35 mm)	0.1	3.5	120.0
SLM 316, as-built *R* = 0.1 [104] (thickness = 10 mm)	0.1	3.92	80.0
SLM 316, as-built and HIP, *R* = −1 [104] (thickness = 10 mm)	0.1	4.00	46.0
LENS 316, as-built, Parallel [105] (thickness = 10 mm)	0.1	6.5	55.0
LENS 316, as-built, Perpendicular [105] (thickness = 10 mm)	0.1	8.0	55.0
LAM Aermet 100 *R* = 0.1 [106] (thickness = 4.0 mm)			
Aermet 100 CG *R* = 0.1	0.1	1.5	400.0
Aermet 100 FG *R* = 0.1	0.1	3.0	400.0
Aermet 100 FG-HRA *R* = 0.1	0.1	4.0	350.0
LPBF 17-4PH from [107] (thickness = 6 mm)			
LPBF 17-4PH, Heat-treated Set 1 crack is parallel to the build direction			
LPBF 17-4PH 1a *R* = 0.1	0.1	0.7	39.0
LPBF 17-4PH 1b *R* = 0.1	0.1	0.1	33.5
LPBF 17-4PH 1d *R* = 0.1	0.1	1.0	33.0
LPBF 17-4PH, Heat-treated Set 2 crack is 90 degrees to the build direction			
LPBF 17-4PH 2a *R* = 0.1	0.1	0.2	35.0
LPBF 17-4PH 2c *R* = 0.1	0.1	0.2	37.5
LPBF 17-4PH 2d *R* = 0.1	0.1	0.2	37.0
LPBF 17-4PH from [108] * (thickness = 6 mm)			
LPBF 17-4PH AB CPCA *R* = 0.1	0.1	5.0	75.0
LPBF 17-4PH AB CPCA *R* = 0.4	0.4	4.0	75.0
LPBF 17-4PH AB CPLRCA *R* = 0.7	0.7	2.0	75.0
LPBF 17-4PH AB *K_max_* = 23	-	2.0	75.0
LPBF 17-4PH HT CPCA *R* = 0.1	0.1	3.0	67.0
LPBF 17-4PH HT CPCA *R* = 0.4	0.4	0.1	67.0
LPBF 17-4PH HT CA *R* = 0.7	0.7	4.0	67.0
LPBF 17-4PH *K_max_* = 25.7	-	2.0	67.0
As-built, WAAM Super Duplex [109] (thickness = 12.7 mm)			
As-built, WAAM Super Duplex TM direction	0	8.25	400.0 **
Ibid	0.2	6.9	400.0
Ibid	0.5	5.5	400.0
As-built, WAAM SSDS LM direction [109] (thickness = 12.7 mm)			
Ibid	0	10.0	350.0 **
Ibid	0.2	7.5	350.0
Ibid	0.5	6.0	350.0

* AB = as-built, HT = heat-treated, CPCA = compression pre-cracking followed by constant amplitude fatigue, CPLRCA = compression pre-cracking followed by load reduction fatigue followed by constant amplitude loading, CA = ASTM E647-23b standard pre-cracking followed by constant amplitude loading. ** The exceptionally high values of the apparent fracture toughness and the fact that the *da/dN* versus Δ*K* curves lie to the right of the curves associated with conventionally built 4340 steel suggests that these specimens had a large residual compressive stress.

**Figure 1:**
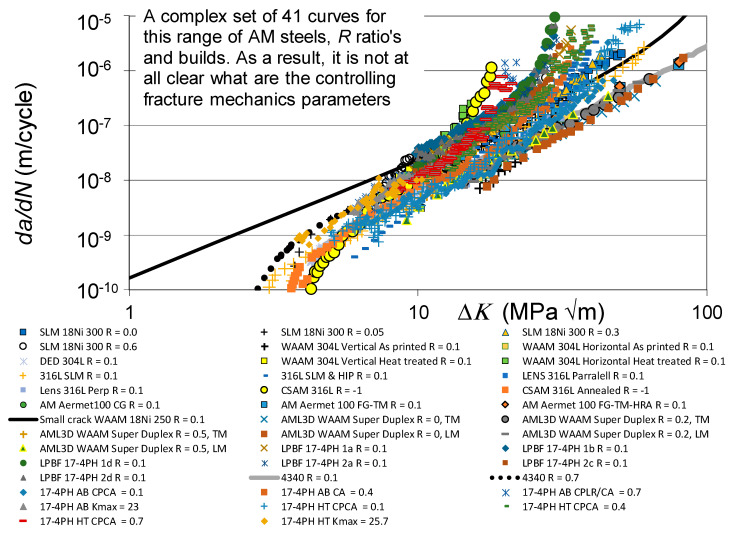
The *da/dN* versus Δ*K* curves associated with crack growth in the tests on the various AM steels described above together with the corresponding curves for tests on the CSAM 361L steel described above.

**Figure 2:**
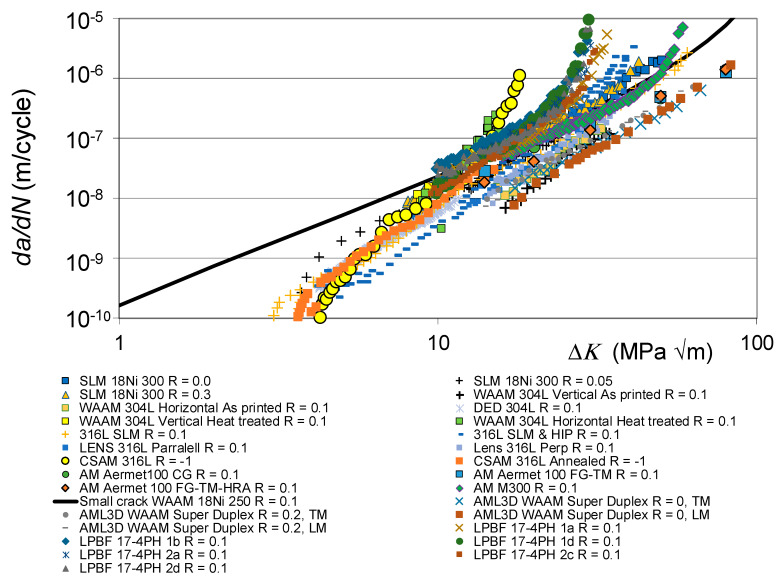
The low *R*-ratio *da/dN* versus Δ*K* curves shown in Figure 1.

**Figure 3:**
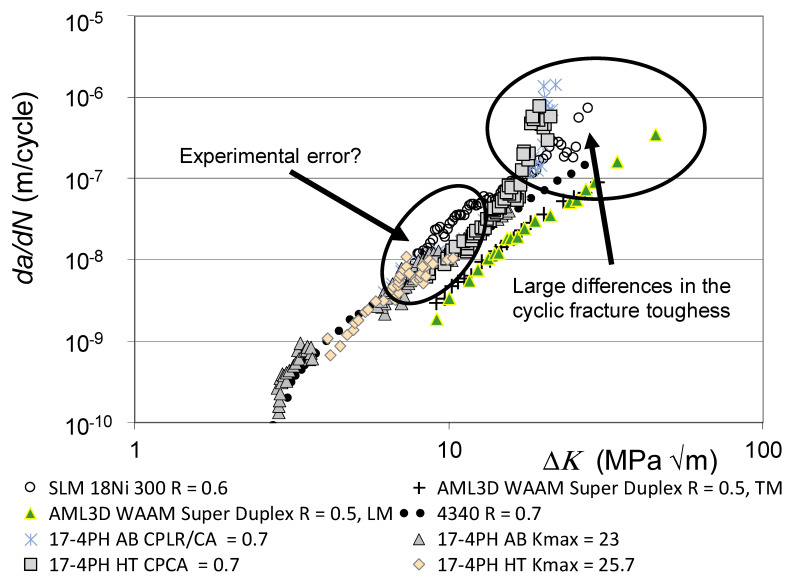
The high *R*-ratio and *K_max_ da/dN* versus Δ*K* curves shown in Figure 1.

The *da/dN* versus Δ*K* curves associated with these various fatigue tests are shown in Figure 1, together with the *R* = 0.1 and 0.7 curves given in [110] for conventionally built high-strength 4340 steel. To help clarify this picture, Figure 2 presents the low *R*-ratio curves, together with the *R* = 0.1 curves obtained for tests on the 4340 steel. Similarly, Figure 3 presents the high *R*-ratio curves as well as the *R* = 0.7 curves obtained for tests on the 4340 steel.

As discussed in [48,111], the fracture toughness and fatigue thresholds associated with AM materials can be significantly different to those associated with the conventionally built material. Indeed, the differences in the apparent fracture toughness associated with these various tests is apparent in Figure 2 and Figure 3. These differences are consistent with those given in the paper by Ritchie [6] on the effect of different heat treatments on both microstructure and on the *da/dN* versus Δ*K* curves associated with the high-strength aerospace steel 300 M. In this study, i.e., in [6], it was shown that varying the temper from one hour at 350 °C to one hour at 650 °C resulted in the plane strain fracture toughness varying from approximately 35 MPa √m to more than 100 MPa √m. The *R* = 0.05 fatigue threshold varied from approximately 2.6 to 8.5 MPa √m.

**Figure 4:**
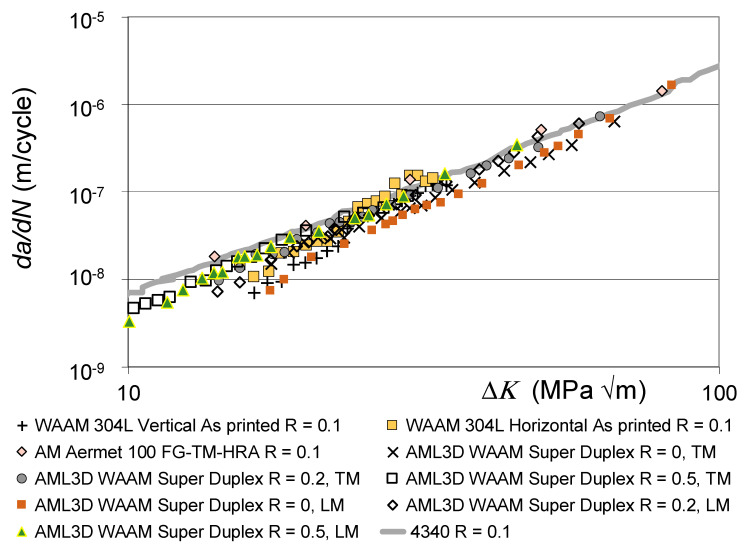
The *da/dN* versus Δ*K* curves associated with AM tests that were to the right of the *R* = 0.1 *da/dN* versus Δ*K* curve associated with the 4340 steel.

Figure 2 and Figure 3 also reveal that there are a few AM tests where their *da/dN* versus Δ*K* curves were to the right of the *R* = 0.1 *da/dN* versus Δ*K* curve associated with 4340 steel (see Figure 4). These curves are associated with AM steels that were left in the as-built state; i.e., they were not heat treated. It is suggested that this phenomenon may be due to the residual stress field that resulted from the build process. This raises the question of whether or not to heat treat limited-life AM replacement parts. This question will be discussed in Section 3.

**Figure 5:**
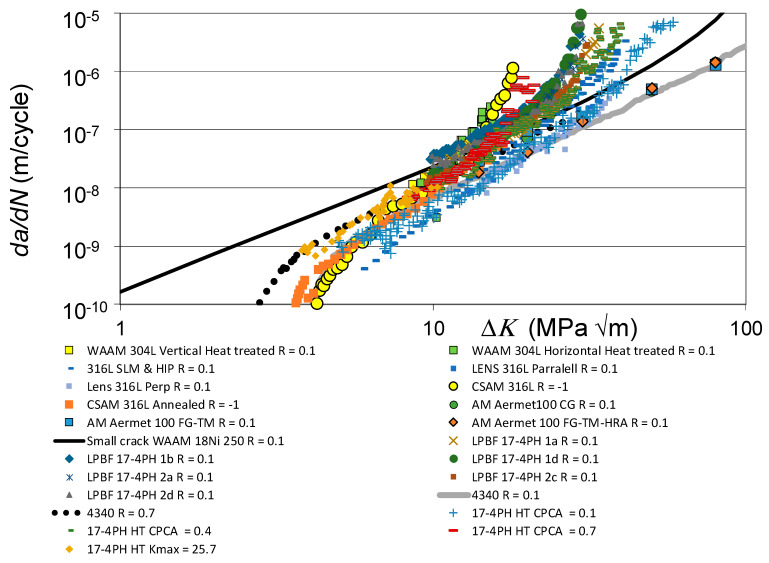
The *da/dN* versus Δ*K* curves shown in Figure 1, albeit with the as-built curves removed.

**Figure 6:**
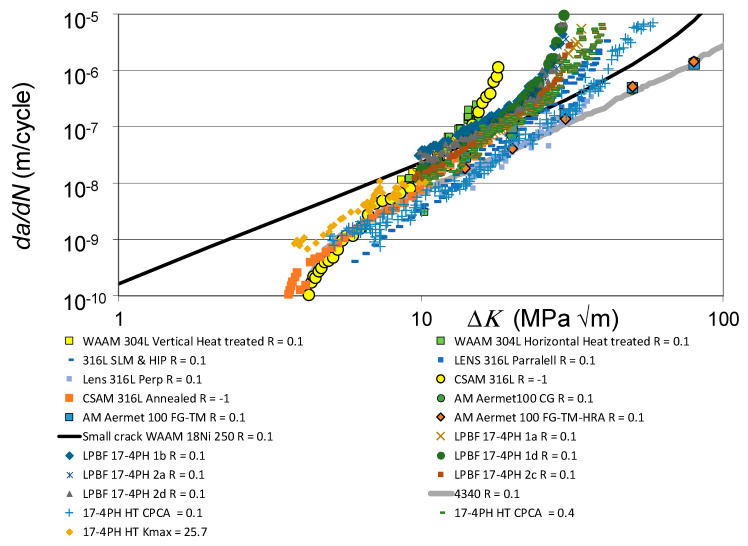
The low *R*-ratio *da/dN* versus Δ*K* curves, albeit with the as-built curves removed.

This raises the question of the suitability of plotting the *da/dN* versus Δ*K* curves associated with as-built and stress-relieved curves on the same graph. To this end, Figure 5 and Figure 6 present the same information contained in Figure 1 and Figure 2, but with curves associated with tests where the specimens were left in the as-built state removed. Whilst, as can be seen in Figure 5 and Figure 6, omitting tests on specimens that were left in the as-built condition reduces the scatter in the data, the relationship between the underlying fracture mechanics parameters and the variability in the crack growth curves is still unclear.

As previously noted, the review papers [37,111] suggest that the fatigue performance of AM steels could be captured by expressing *da/dN* as a function of Δ*κ*. Based on these studies, it is hypothesised that the crack growth curves associated with this range of AM steels will have similar *da/dN* versus Δ*κ* curves. The present paper uses the Total Least Squares method outlined in [55,86] to determine the constants Δ*K_thr_* and *A* associated with each of these forty-plus tests and thereby evaluate this hypothesis.

**Figure 7:**
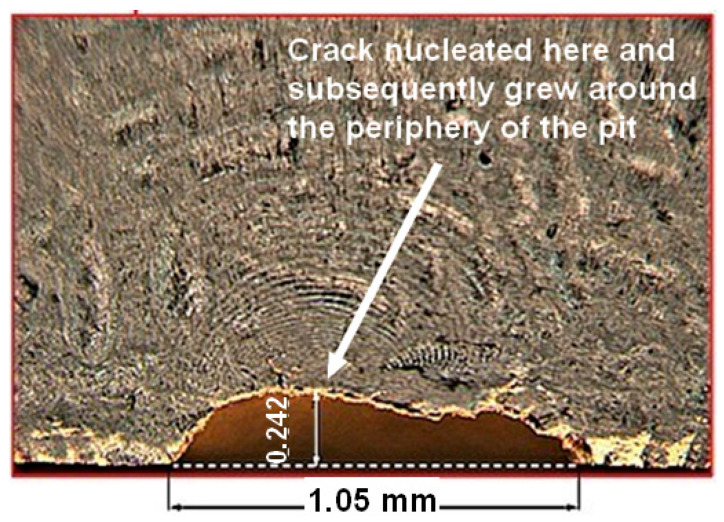
An example of how a 3D crack can nucleate at the bottom of a surface defect/pit before evolving into a near semi-elliptical shape, all dimensions are in mm. In this instance, the specimen was a Boeing Space, Intelligence, and Weapon Systems laser-powder-built (LPBF) Scalmalloy^®^ part.

It was previously mentioned that USAF Structures Bulletin EZ-SB-19-01 [5] requires the fatigue thresholds to be determined. In the case of a durability analysis of a limited-life AM/CSAM part, this means an estimate for the fatigue threshold associated with naturally arising 3D cracks that nucleate from porosity/lack of fusion or a corrosion pit. As can be seen in Figure 7, which shows how cracks nucleated and subsequently grew from the base of a surface-breaking defect in a BSI&WS AM Scalmalloy^®^ specimen, this raises the question of how to determine an estimate for the stress intensity factors associated with naturally occurring 3D cracks that nucleate at the bottom of a surface-breaking porosity, or an etch pit, in an AM/CSAM part. Furthermore, this solution is also required to determine the worst-case *da/dN* versus Δ*K* curve that is needed for the durability assessment of limited-life AM and CSAM replacement parts. Figure 7 illustrates that such naturally occurring cracks often first grow around the periphery of the porosity/pit and only subsequently transform into what is essentially a semi-elliptical surface crack. This phenomenon, which is not confined to AM materials, is also highlighted by Cheng et al. [112].

Given the complexity involved in performing the detailed three-dimensional finite element analyses needed to correctly tackle this problem, Burns et al. [88] noted that, when the crack depth is small in comparison to the dimensions of the pit, it is often modelled as a semi-elliptical surface crack. In this approach, for a uniform remote stress, the stress intensity factor at the deepest point of the crack is estimated using the Newman–Raju equations, with the crack depth assumed to be the pit depth plus the distance from the periphery of the crack. In [88], this approach was referred to as the “periphery method”. As a result, a secondary focus of this paper is to illustrate that using the Murakami and periphery methods to determine the stress intensity factors associated with small 3D cracks that nucleate at the base of a pit is not necessarily accurate. This conclusion is achieved by first developing a simple approximate analytical formula for the stress intensity factor associated with a large specimen with a naturally occurring semi-circular crack that emanates from the bottom of a hemi-spherical surface defect, where the depth of defect is small in comparison with the dimensions of the structure, and the depth of the crack is much smaller than the dimensions of the defect from which the crack has nucleated. This formula is validated using both conventional three-dimensional finite element analysis and the three-dimensional finite-element-alternating technique. Details on the 3D finite-element-alternating technique can be found in [113,114,115,116,117,118,119,120,121,122] and in Appendix A. This analytical solution will (hopefully) enable improved estimates for the fatigue threshold associated with three-dimensional cracks that nucleate from a surface-breaking lack of fusion or pitting in AM/CSAM parts.

**Figure 8:**
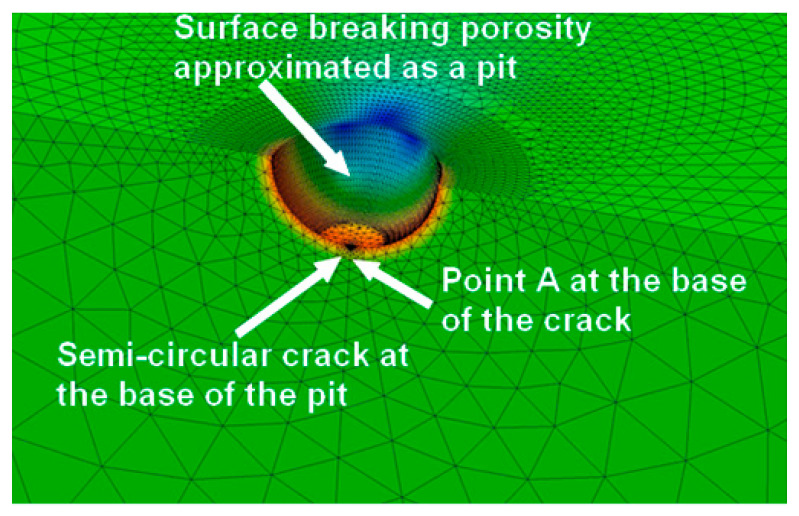
Close-up view of the finite element mesh used in the Zencrack^®^ analysis of a small crack that emanates from the bottom of a 0.5 mm radius hemi-spherical pit in a large (10 mm deep) block uncracked pit.

To illustrate problems that can arise when people use the Murakami and the periphery methods to estimate the stress intensity factors associated with small naturally occurring cracks that nucleate from a pit, let us consider the case of a 0.01 mm deep semi-circular crack that emanates from the base of a 0.5 mm deep hemi-spherical pit in a large block of 7050-T7451 aluminium alloy that was 50 mm long, 20 mm wide, and 10 mm deep (see Figure 8). The pit depth was chosen since it is reasonably representative of that seen in Figure 7 for AM specimens with pitting/surface breaking porosity/lack of fusion, as well as for pits seen in operational aircraft [123,124]. On the other hand, the depth of the crack that emanated from the periphery of the defect was based on the statement given in [29] that “typical initial discontinuity sizes are about equivalent to a 0.01 mm deep fatigue crack”. The block was assumed to be subjected to a remote uniaxial stress of 300 MPa that was acting in the length direction.

The computed values of the stress intensity factor were used to examine the accuracy of a range of approximate expressions that are used to estimate the maximum value of the stress intensity factor *K_max_*.

## 3. Results

**Figure 9:**
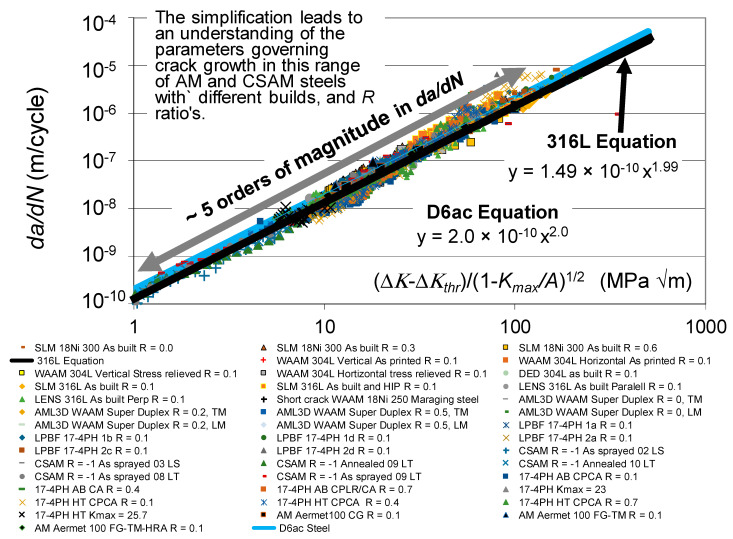
The *da/dN* versus Δ*κ* relationship associated with these forty-one AM and CSAM crack growth curves.

Let us first address the following question: Can the crack growth in the cross-section of AM steels outlined in Section 2 be captured by allowing for the variability in the fatigue threshold (Δ*K_thr_*) and the apparent cyclic toughness (*A*)? To this end, Figure 9 presents the same data shown in Figure 1 but with *da/dN* now plotted as a function of Δ*κ*. For comparison purposes, Figure 9 also contains the *da/dN* versus Δ*κ* curve, given in [41] for conventional and additively manufactured 316L steels and in [21] for D6ac steel.

**Figure 10:**
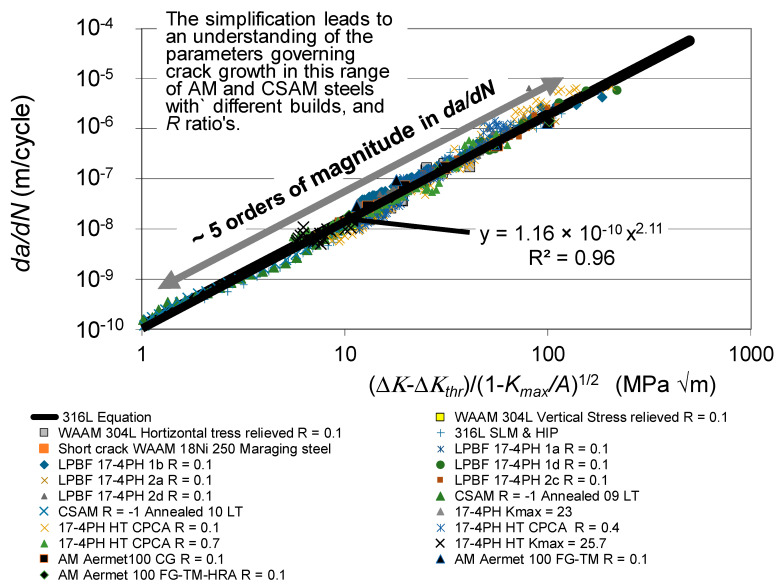
The *da/dN* versus Δ*κ* relationship associated with the AM and CSAM crack growth curves shown in Figure 9, albeit with the curves associated with as-built specimen tests omitted.

As discussed in Section 2, the build process can result in significant residual stresses, and these stresses can result in a distorted *da/dN* versus Δ*K* curve. Indeed, Kundu et al. [125] illustrated that when using the Hartman–Schijve crack growth equation, i.e., Equations (1) and (2), to accurately predict crack growth in AM parts, it is necessary to know the effect of the residual stresses on the value of the *K_max_*. Unfortunately, none of the papers presented the residual stress field. As a result, Figure 10 presents the same data shown in Figure 9, albeit with the data associated with specimens tested in the as-built state omitted.

Figure 10 reveals that when this is performed then, to a first approximation, not only do the crack growth curves simplify, but each of the different curves also have similar *da/dN* versus Δ*κ* relationships. Furthermore, this relationship holds over approximately five orders of magnitude in *da/dN*. In other words, to a first approximation, the differences in the *da/dN* versus Δ*K* curves associated with these various AM and CSAM steels are essentially due to the differences in the fatigue thresholds and the apparent cyclic toughness. The values of Δ*K_thr_* and *A* associated with each of these tests are given in Table 1.

## 4. Implications for Limited-Life AM and CASM Replacement Parts

As previously noted, the USAF Structures Bulletin EZ-SB-19-01 [5] states that the airworthiness certification of a limited-life AM part requires a durability analysis. To the best of the authors’ knowledge, the 1999 USAF-Boeing study [126] was the first to highlight that the durability analysis should use a valid small crack *da/dN* versus Δ*K* curve. Indeed, NASA-HDBK-5026 [127], which addresses the certification requirements for AM space vehicle parts, mandates the use of the worst-case (upper-bound) *da/dN* versus Δ*K* curve. (NASA-HDBK-5010 [128], which addresses the certification requirements for conventionally built space vehicles parts, also mandates the use of the worst-case *da/dN* versus Δ*K* curve.) This requirement means that for each AM build process, there should be a statistically significant number of repeat *da/dN* versus Δ*K* test curves and that these curves must be characterised in such a fashion that the worst-case *da/dN* versus Δ*K* test curve can be computed. Whilst this has been achieved for AM Ti-6Al-4V [10,48,129], no similar studies have been performed for AM steels. This shortcoming needs to be addressed. In [10,129], this worst-case curve was determined using the Hartman–Schijve equation.

As previously mentioned, USAF Structures Bulletin EZ-SB-19-01 [5] requires an estimate for the fatigue threshold. Such worst-case estimates are also lacking. In the case, on AM Ti-6Al-4V, it was shown [10,129] that this can be achieved using the Hartman–Schijve crack growth formulation. A key feature of [10,48] is that, as discussed in [129], the worst-case threshold was estimated to be approximately 0.1 MPa √m. (Given the crack length versus cycles histories in these studies, there was no alternative to determining the worst-case threshold in this fashion. This point is discussed in more detail in the next paragraph).

As is evident in Table 1, the present review reports a similar “worst-case” threshold for the AM steels studied. However, many of the studies evaluated in this paper used the load reducing test protocol outlined in the fatigue test standard ASTM E647-23a. Unfortunately, it has long been known [110,129,130] that this test protocol can result in invalid data beneath crack growth rates of approximately 10^−8^ m/cycle. As a result, the error levels associated with several of the tests examined is uncertain. Similarly, using ASTM compact tension test specimens, as was performed in most of the studies, makes it difficult to independently substantiate the supposition that the worst-case fatigue threshold is so low. Fortunately, as explained in [129], this independent check can be performed by using single-edge notch tension (SENT) test specimens that are loaded using hydraulic grips. The stress intensity factor solution for this specimen test configuration, which is given in [131], means that if the worst-case threshold is so low and if crack growth is governed by Equations (1) and (2), then the crack growth history will be exponential. It also means that the worst-case crack growth equation needed for a damage tolerance assessment and the for a durability assessment will coincide, see [131] for more details. However, it must be stressed that, as explained in [48], it is important not to use the stress intensity factor given in the test standard ASTM E647-23a for SENT specimens. This is because this solution is for a pin-loaded test rather than for a specimen that is loaded via hydraulic grips.

Returning to the question of durability, the assessment of AM materials, whilst it is tempting to believe that the (durability) ranking of materials and processes can be achieved via tests performed in accordance with the main body of the ASTM E647-23b fatigue test standard [24], the paper by Venkateswara Rao, Yu, and Ritchie [132] revealed that the ranking of the fatigue performance of materials based on tests performed on long cracks does not necessarily correspond to the ranking associated with the growth of small cracks. This observation is consistent with the statements contained in Appendix X3 of ASTM E647-23b:

“Fatigue cracks of relevance to many structural applications are often small or short for a significant fraction of the structural life. The growth rates of such cracks usually cannot be measured with the standard procedures described in the main body of Test Method E647”.

As such, tests on naturally occurring 3D cracks are essential for assessing/ranking the build and post-processing procedures required for AM and CSAM parts in order to meet the operational life requirements for a limited-life replacement part. Indeed, this statement holds regardless of whether the part is built from AM steel, AM Ti-6Al-4V, AM Inconel 718, or an AM aluminium such as LPBF Scalmalloy^®^, etc.

In this context, it should be noted that Section 2 revealed that different build processes and heat treatments can yield large differences in the apparent cyclic fracture toughness’s and hence in the *da/dN* versus Δ*K* curves. On the other hand, it would appear that, to a first approximation, these differences are significantly reduced if *da/dN* is expressed as a function of Δ*κ*. The Introduction also noted that, to a first approximation, an estimate of the worst-case *da/dN* versus Δ*K* curves associated with naturally occurring 3D cracks in AM materials could often be estimated from the long crack *da/dN* versus Δ*κ* curve by setting the fatigue threshold term to a small, near-zero value, typically 0.1 MPa √m. Thus, for any given material and build process, it would appear that, to a first approximation, the durability assessment of an AM/CSAM part is primarily reflected by the variability of just one fracture mechanics parameter, namely the cyclic fracture toughness (*A*), on the operational life of the part to be considered. Of course, the level of porosity/lack of fusion and residual stresses can also affect durability. However, these are not fracture mechanics parameters. Fortunately, the combined effect of porosity/lack of fusion and build/post-build procedures will be apparent from tests on specimens where cracking is allowed to nucleate and (subsequently) grow naturally, i.e., without the introduction of artificial starter cracks.

In this context, it should be noted that, as explained in [45], the operational life of an AM, or a CSAM limited-life part, does not need to be equal to that of the design life of the part. It only needs to be sufficiently attractive to be worth implementing. In other words, as was first highlighted in [45], the build protocol, the level of post-manufacturing treatment, the acceptable level of porosity, and the allowable level of surface roughness are functions of operational life requirement of the AM/CSAM (limited-life replacement) part, the geometry of the part, the operational flight loads, and the operational environment seen by the part. In other words, there is “no one-size-fits-all” answer to these questions. Consequently, the observation that, for the AM and CSAM steels examined in this study, the *da/dN* versus Δ*κ* curves are similar supports prior statements, which were associated with studies into AM Ti-6Al-4V parts [45], about the potential for using fracture toughness measurements in conjunction with the flight load spectrum and the operational life requirement to guide the choice of the AM build process, the associated post-manufacture treatment, the acceptable level of porosity, and the acceptable level of surface roughness.

Recalling that Section 5 of MIL-STD-1530Dc [11] states that the role of testing is to correct/validate the analysis, this means that this process, i.e., the use of an LEFM-based durability assessment to guide the choice of the build process and the level of post-build processing, is an essential part of the airworthiness acceptance of a limited-life AM/CSAM replacement part. That said, as has previously been stated, the performance of the part when subjected to the anticipated operational environment should also be a major consideration.

### On the Question of Heat Treatment

We had previously seen that there were cases where it would appear that, when left in the as-built condition, the *da/dN* versus Δ*K* curve appeared to be to the right of the curves for conventionally built (high-strength) 4340 steel. In these instances, it was conjectured that this observation was likely to be due to the large residual stress that resulted from the build process. This observation would appear to suggest that leaving an AM part in the as-built condition could be beneficial. However, the in-service cracking seen in the upper (compressive) surface of the D6ac wing pivot fitting of F111 aircraft in service with the Royal Australian Air Force (RAAF) [133] highlighted the problems associated with a large residual compressive stress.

To clarify this statement, it should be noted that in order to keep flying the F-111, it was subjected, every five years, to a cold proof load test (CPLT). As part of the CPLT, the airframe was subjected to (wing) loads that ranged from 7.33 g to −3.4 g, which meant that the upper (compressive) surfaces of the wing saw tensile stresses were equivalent to 3.4 to −7.3 g loads. However, the stress concentrator at stiffener runout number 2 in the upper-wing pivot fitting was such that after unloading from a −7.3 g (compressive) load, the local region was left with a large tensile residual stress. Consequently, during operational service, the tensile flight loads seen on the upper surface of the wing-nucleated cracks subsequently grew [133]. These cracks resulted in catastrophic failures of several F111 wings during subsequent CPLT. Since this region was at a nominally compression-dominated location, it was not flagged, at either the design stage or following full-scale fatigue testing, as being fatigue critical. Consequently, a non-destructive inspection (NDI) programme was not developed for this location, and failure during CPLT was unexpected. A more detailed, solid, mechanics-based discussion on this topic can be found in [134].

Since a great deal of the impetus for the use of limited-life AM/CSAM parts is associated with the problem of corrosion damage, and since corrosion damage is more acute on the upper (compressive) surface of a wing, the problem is that the compressive flight loads can add to the existing residual compressive stresses (due to the build), so that, if the residual stress is large enough, then when unloading, the part can be left with a residual tensile stress. In such instances, the tensile flight loads experienced on the upper surface of the wing can nucleate cracks, which may, as per the F111 experience, grow. Since the potential for a nominally compressively loaded part to be fatigue critical would not have been identified at the design stage, or in the full-scale fatigue test, an NDI programme would not have been developed for this part, and cracking would only be detected once it had become large.

Consequently, whilst leaving a part in the as-built condition may, at first glance, appear to be attractive, this conclusion does not necessarily follow. Indeed, as mentioned above and as aptly illustrated in [21], which analysed an early F-111 wing failure, and in [45], which analysed failure in a US Navy F/A-18 centre barrel test, the accurate prediction of cracks that nucleate naturally and then grow to failure under operational flight loads requires the use of a valid (worst-case) crack growth curve. (As previously flagged, both of these analyses used Equations (1) and (2)). Furthermore, as noted in Appendix X3 of ASTM E647 [24], this curve differs from that obtained using the test protocol outlined in the main body of ASTM E647. Consequently, the *da/dN* versus Δ*K* curves obtained using the test protocol outlined in the main body of ASTM E647, namely compact tension (CT), single-edge notch tension/bending, middle crack tension, etc., test specimens are of limited use in deciding the post-build treatments that are needed to meet the operational life requirement associated with a limited-life AM replacement part. Of course, this statement is not limited to AM or CSAM steels and applies to any AM/CSAM limited-life part.

**Figure 11:**
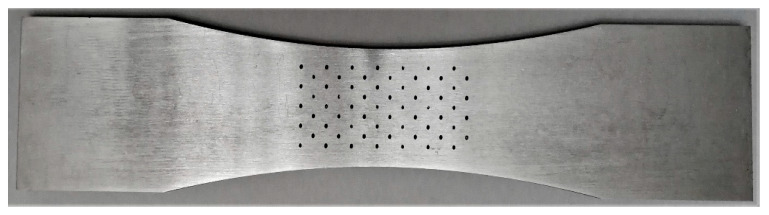
A typical “small crack” test specimen with an array of etch pits. These etch pits act as potential crack nucleation sites.

As such, it would appear that future studies are required to assess the level of heat treatment, HIPing, etc., that are needed to meet the operational life requirements of both AM and CSAM limited-life replacement parts. Such tests must focus on the effect of these post-build processes on the growth of the worst-case, naturally occurring, three-dimensional cracks. In operational aircraft, these worst-case cracks are often referred to as “lead cracks” [26,27,28,29]. When performing laboratory tests, the large variability in the crack growth rates that is associated with cracks that nucleated naturally is such that the “worst-case” *da/dN* versus Δ*K* curve can be determined, as in [15,16,28,89], by using a specimen with an array of possible nucleation sites. One such example is shown in Figure 11. One way to create such pits is by localised etching (see [15,16]).

In the case of AM and CSAM aluminium alloys, the worst-case *da/dN* versus Δ*K* curve can also be determined as in [35] by, prior to the fatigue test, exposing the specimens to a 5% by weight NaCl salt fog in an ASTM B117-19 environmental chamber [64]. The effect of this exposure is to (also) produce a number of corrosion sites that have the potential to nucleate a crack and, as such, for AM and CSAM aluminium alloy specimens, acts in a fashion similar to that described above.

This leads to the question: How do we determine the stress intensity factors for 3D cracks that nucleate at a pit? This question is addressed in the next section.

## 5. Estimating the Fatigue Threshold Associated with Naturally Occurring 3D in AM and CSAM Materials

To illustrate problems that can arise when people use the periphery method to estimate the stress intensity factors associated with small cracks that nucleate from a pit, let us consider the problem outlined in Section 2, viz: a small 0.01 mm deep semi-circular crack that emanates from the base of a small 0.5 mm deep hemi-spherical pit in a large block of 7050-T7451 aluminium alloy that was 50 mm long, 20 mm wide, and 10 mm deep (see Figure 8). As noted in Section 2, the pit depth was chosen since it is reasonably representative of that seen in Figure 7 for AM specimens with pitting/surface-breaking porosity/lack of fusion, as well as for pits seen in operational aircraft [123,124]. On the other hand, the depth of the crack that emanated from the periphery of the defect was based on the statement given in [29]: “typical initial discontinuity sizes are about equivalent to a 0.01 mm deep fatigue crack”.

To this end, let us define the depth of the crack as “a” and the radius of the hemispherical pit as “*r*”. Furthermore, let us assume that *a* << *r*. Let us first assume that, as per the “periphery” approach, the stress intensity factor *K* at point *A*, the deepest point of the crack—see Figure 8—can be modelled as a crack with a depth equal to r + a.

Since we are interested in estimating the stress intensity factor at the deepest point of this small naturally occurring crack, i.e., at point *A* in Figure 8, we will define the value of *K* determined using the periphery method as “*K_periph_*”. Ignoring boundary effects, which is acceptable since the dimensions of the block are large in comparison with both the dimensions of the pit (porosity/lack of fusion) and the (nucleated) crack, the stress intensity factor at point *A* (determined using this approximation) can be expressed as

*K_periph_* = 2*σ*√(π (*r* + *a*))/π (4)

where *σ* is the applied remote stress.

Let us denote the stress concentration factor at the pit/porosity/lack of fusion as *K_T_*. For small values of the ratio *a*/*r*, the actual value of the stress intensity factor (at the deepest point of the crack), defined as *K_true_*, can now be expressed as follows:

*K_true_* = 2 *K_T_ σ* √(π*a*)/π (5)

**Figure 12:**
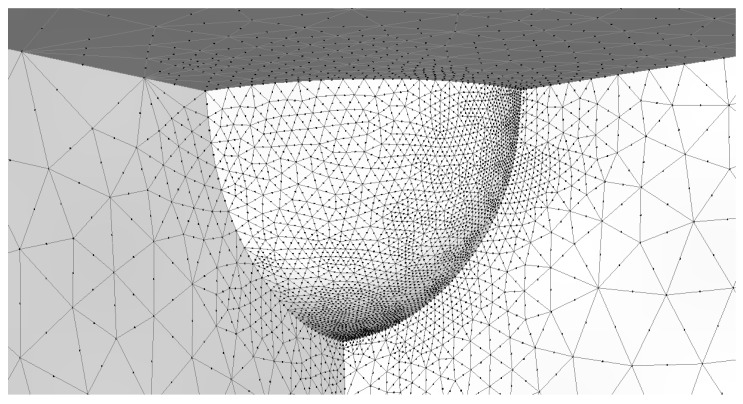
A local view of the finite element mesh associated with the uncracked pit.

Before Equation (5) can be used to compute the stress intensity factor solution, it is necessary to determine the stress concentration factor *K_T_*. Since the radius of the pit is small in comparison to the dimensions of the structure, the closed-form formulae given by An et al. [135] for *K_T_* can be used. This yields a value of *K_T_* = 2.09. This value was validated via a three-dimensional analysis that used the finite element programme AutoCAD Nastran^®^ [136]. The finite element mesh used in this study is shown in Figure 12. (Symmetry considerations meant that only a quarter of the structure needed to be modelled.) This mesh consisted of 31,966 ten-noded iso-parametric tetrahedral elements and 49,955 nodes. The analysis was repeated using Zencrack^®^ in conjunction with the finite element code Abaqus^®^. In the Zencrack^®^ analysis, the mesh consisted of 72,958 ten-noded iso-parametric tetrahedral elements and 106,595 nodes. Both analyses returned a *K_T_* of approximately 2.10, thereby supporting a *K_T_* of approximately 2.1.

A Zencrack^®^ analysis and a finite-element-alternating technique (FEAT) of the problem with a small 0.01 mm semi-circular crack emanating from the bottom of the pit and a remote uniaxial stress of 300 MPa were then performed. The stress acts in the length direction, and all of the other faces are unloaded. (A brief discussion on the history of the finite-element-alternating technique and its application to a range of problems associated with both AM and conventionally manufactured metals, including failures in full-scale fatigue tests, is given in Appendix A. As explained in Appendix A, an advantage of the FEAT approach is that cracks do not need to be explicitly modelled and that the stress intensity factor distribution around the crack front can be computed using only the uncracked finite element model). In the case of the FEAT analysis, two different mesh densities were used and, as a result of symmetry considerations, only a quarter of the structure was modelled. The first is as shown in Figure 12 and described above. The second used a finer mesh that consisted of 111,885 ten-noded iso-parametric tetrahedral elements and 180,971 nodes. The resultant computed values of *K_max_*, which used these two different uncracked meshes, differed by less than 1.5%. The value obtained using the finer of the two meshes, viz 2.32 MPa √m, is given in Table 2. Table 2 reveals that the FEAT solution differs from that computed using the analytical solution, i.e., Equation (5), by approximately 3%.

**Figure 13:**
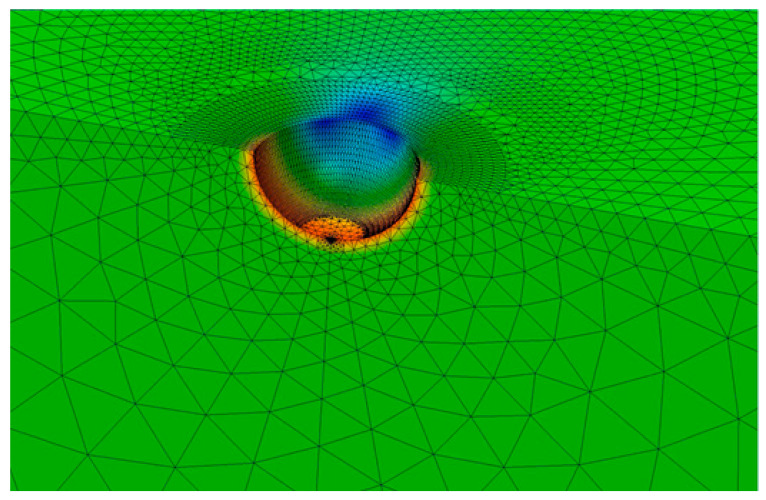
A local view of the finite element mesh associated with the Zencrack^®^ analysis.

The Zencrack^®^ analysis also used two different mesh densities. One mesh consisted of 87,578 ten-noded iso-parametric tetrahedral elements and 135,590 nodes. The other consisted of 167,269 ten-noded iso-parametric tetrahedral elements and 106,538 nodes. As is standard practice in such analyses, the mid-side nodes close to the crack tip were moved to the ¼ points. A local view of the finite element mesh associated with the coarser mesh is shown in Figure 13. The resultant values of *K_max_* differed by less than 1.5%. The value obtained using the finer of the two meshes, viz 2.25 MPa √m, is also given in Table 2. In this instance, the difference between the finite element solution and that obtained using Equation (5) is less than 1%. This study suggests that each of these three different methods was able to estimate reasonably well the stress intensity factor solution for this problem.

The errors associated with Equation (3), i.e., Murakami’s formulae, and Equation (4), the periphery method, to estimate the stress intensity factor are presented in Table 3. Here, it can be seen that the values of *K_max_* obtained using the Murakami and the periphery approaches are approximately 8.7 and 7.6 MPa √m, respectively. Both Equations (3) and (4) significantly overestimated the stress intensity factor. Consequently, using either approach to estimate the fatigue threshold associated with naturally occurring 3D cracks that emanate from surface-breaking porosity/lack of fusion is not recommended.

The analysis was then repeated for a 0.1 mm deep hemispherical pit with a 0.01 mm deep semi-circular crack emanating from the deepest point of the pit. This pit depth is also reasonably representative of that seen in Figure 7 and in operational aircraft [123,124]. Since the dimensions of the pit and the crack are small with respect to the dimensions of the block, Equation (5) returns the same value for the stress intensity factor, viz, 2.24 MPa √m. The FEAT analysis and the Zencrack^®^ finite element analysis (both) gave values of approximately 2.1 MPa √m. This value differs from the value obtained using the analytical solution, i.e., Equation (5), by approximately 5%. In other words, the maximum value of the stress intensity factor was relatively independent of the cross-sectional area of the pit/surface breaking porosity.

In contrast, the Murakami approximation (Equation (3)), which is strongly dependent on the cross-sectional area of surface-breaking porosity/pit, returns a value of 3.88 MPa √m. This value differs from the values given by the finite element solution and the value obtained using Equation (5) by approximately 85% and 73%, respectively. The value obtained using Equation (3), the periphery approximation, is 3.55 MPa √m. This value also differs significantly from values obtained using Equation (5) and by the two different finite element analyses.

Of course, the value of the stress intensity factor obtained using Equation (5) and the finite element analysis is strongly dependent on the size of the (assumed) crack. For example, for a 0.1 mm deep hemispherical pit with a 0.002 mm deep semi-circular crack emanating from the deepest point of the pit, the analytical solution (Equation (5)) returns a value of 1.01 MPa √m. The FEAT and the Zencrack^®^ finite element solutions return values of 1.04 and 1.00 MPa √m, respectively. In this case, the value obtained using Equation (3), the Murakami approximation, is 3.87 MPa √m, and the value obtained using Equation (4), the periphery approximation, is 3.42 MPa √m. Both approaches significantly overestimate the value of the stress intensity factor.

As such, when performing the necessary LEFM durability assessment of an AM limited-life replacement part, and when estimating the fatigue threshold associated with naturally occurring cracks that nucleate from porosity/lack of fusion/corrosion pits, it is generally best, when the size of the initial crack being analysed is less than (or comparable to) the depth of the porosity/lack of fusion/pit from which the crack nucleates, to account for the shape of the porosity/lack of fusion/pit. Details on how to perform such an analysis can be found in [16,30].

In other words, it is suggested that the Murakami approximation and the periphery method should not be used to assess the fatigue threshold associated with naturally occurring 3D cracks in AM/CSAM materials or in the durability assessment of limited-life AM/CSAM replacement parts. That said, if as is hypothesised in [137], the level of porosity can be significantly reduced by a newly developed Net-AM processing technique, then the durability analysis of an AM/CSAM part may well be simplified.

Unfortunately, there are currently no studies that relate the level of porosity in an AM part to the “build quality” as defined by the USAF [138,139]. This shortcoming needs to be addressed.

## 6. Conclusions

It would appear that, to a first approximation, for the steels discussed, the effect of different yield stresses, build processes (AM, CSAM, or conventional), annealing temperatures, and *R* ratios on the *da/dN* versus Δ*K* curves can often be captured by accounting for the associated changes in the fatigue threshold and the apparent cyclic toughness. Furthermore, it appears that, in each case, there is a (near) power law *da/dN* relationship Δ*κ* that holds over approximately five orders of magnitude in *da/dN*.

However, whilst these forty-two tests that range from AM steels to CSAM 316L steel and the (approximately) one hundred tests on AM and conventionally manufactured Ti-6Al-4V, AM Inconel 718, AM Inconel 625, LPBF built Scalmalloy^®^, and CSAM built pure metals reported in the open literature, which also revealed a near power law *da/dN* relationship Δ*κ*, go a long way to making a point, they are NOT mathematical proof. They are merely empirical evidence. Nevertheless, this observation is quite compelling.

It is also suggested that the observation that, for the heat-treated (annealed) AM steels and the CSAM 316L steel examined in this review, the *da/dN* versus Δ*κ* curves are similar supports prior studies into crack growth in AM Ti-6Al4V that suggested using fracture toughness measurements in conjunction with the flight load spectrum and the operational life requirement to guide the choice of the AM/CSAM build process and the associated post-build heat treatment needed for an AM limited-life replacement part. That said, the performance of the part when subjected to an aggressive maritime environment should also be a major consideration.

It should be noted that, whilst this paper has largely focused on AM steels, the problem of corrosion damage in operational aircraft is a major concern [140]. Consequently, the ability of limited-life AM parts to alleviate sustainment problems associated with the corrosion of aluminium alloy airframes is particularly important. In this context, it should be noted that the US Navy-funded review [141] reported that of all of the additively manufactured (AM) aluminium alloys evaluated, Scalmalloy^®^, which is an Al-Sc-Mg alloy, had the most attractive mechanical properties. The subsequent papers [15,35] found that not only was the heat-treated Boeing Space-Intelligence and Weapon Systems LPBF-built Scalmalloy^®^ largely resistant to corrosion, but that its damage tolerance was also superior to that of conventionally manufactured 7075-T6, and its durability, both with and without prior exposure to an aggressive environment, could be predicted using the Hartman–Schijve crack growth equation. As such, the observations outlined in this paper are not confined to AM/CSAM steels and, as such, have implications for the implementation and certification of limited-life AM parts for a wide range of airframe materials.

It is also shown that, when estimating the fatigue threshold associated with naturally occurring cracks that nucleate from porosity/lack of fusion or surface pitting, it is generally best to model (account for) the shape of the porosity/lack of fusion/pit. It is also suggested that the Murakami approximation and the periphery method should not be used to assess either the fatigue threshold associated with naturally occurring 3D cracks in AM/CSAM materials or the durability assessment of limited-life AM/CSAM replacement parts.

## Figures and Tables

**Table 2:** Comparison of the computed values for *K*_max_ (MPa √m) and the value obtained using Equation (5).

From the FEAT Analysis	From the Zencrack^®^ Analysis	Equation (5)
2.32	2.25	2.25

**Table 3:** Comparison of the computed values for *K*_max_ (MPa √m) for a 0.5 mm deep hemispherical pit with a 0.01 mm deep semi-circular crack.

Approximate Formulation	Estimated Value of *K*_max_ (MPa √m)	% Error
Equation (3) (Murakami’s formulae)	8.7	287
Equation (4) (the periphery approach)	7.6	238

## Data Availability

No new data were created or analyzed in this study. Data sharing is not applicable to this article.

## Appendix A. The Finite-Element-Alternating Technique (FEAT)

The three-dimensional finite-element-alternating technique (FEAT) was developed in the early 1980s [113]. It is a development of the analytical solution given in [114] for a three-dimensional crack subjected to arbitrary loads. It has the advantage that, when computing the stress intensity factor distribution around the crack front, the crack is not modelled explicitly. Consequently, regardless of the shape of the crack and the applied loads, only the uncracked finite element model is needed. This technique has been widely used for assessing the structural integrity of both conventionally and additively manufactured structures [15,16,21,30,35,43,45,113,115,116,117,118,119,120,121,122]. This includes crack growth in an early F-111 wing failure [21] and in a US Navy F/A-18 centre barrel fatigue test [45].

Here, it should be noted that the F-111 wing test analysed in [21] was chosen due to the central role that the F-111 has in the development of current airworthiness certification requirements (see [142]) and in the role that this test had in the acceptance of the F-111 into operational service, see [21].

A detailed review of the three-dimensional finite-element-alternating technique can be found in [120].
